# Impact of Dance or Music and Meditation on the Progression of Parkinson Disease With Mild or Moderate Severity: Protocol for a Pilot Randomized Controlled Trial

**DOI:** 10.2196/59018

**Published:** 2024-10-29

**Authors:** Bhagyashree Mehrotra, Neha Rai, Rajani MR, Aparna Budhakar, Ritika Aggarwal, Raj Vinodkumar Agarbattiwala, Mona Thomas, Sampada Patole, Paresh Doshi

**Affiliations:** 1 Stereotactic and Functional Neurosurgery Department Jaslok Hospital and Research Centre Mumbai India; 2 Department of Anaesthesiology Jaslok Hospital and Research Centre Mumbai India; 3 Psychology Department Jaslok Hospital and Research Centre Mumbai India

**Keywords:** music therapy, dance therapy, neurodegenerative disorders, meditation, quality of life, music, pilot study, Parkinson disease, well-being

## Abstract

**Background:**

Parkinson disease (PD) is a progressive neurodegenerative disorder characterized by motor dysfunctions and nonmotor symptoms. Current treatments do not alter disease progression, highlighting the need for alternative therapies. Music, dance, and mindfulness meditation have shown the potential to improve symptoms and quality of life in patients with PD.

**Objective:**

This study aims to evaluate the effectiveness of dance or music and meditation on PD progression, cognitive functions, mood, behavior, and caregiver burden.

**Methods:**

This study is a single-blinded, longitudinal, parallel, randomized controlled trial. The participants consist of 30 patients with mild to moderate PD residing in Mumbai, India, who can physically participate in the activities. The exclusion criteria include advanced PD, severe balance issues, age >80 years, and other movement disorders. Participants in the intervention group will engage in dance or music sessions and guided meditation thrice weekly for 6 months. The control group will continue their usual activities and medication. The primary outcome is the progression of PD symptoms, measured using the Unified Parkinson’s Disease Rating Scale I-III, and quality of life, measured using the Parkinson’s Disease Questionnaire-39. The secondary outcomes include cognitive functions (Mini-Mental State Examination), mood (Beck Depression Inventory and Parkinson Anxiety Scale), mobility (timed up and go and Berg Balance Test), behavioral disorders (Questionnaire for Impulsive-Compulsive Disorders in Parkinson’s Disease Rating Scale), and caregiver burden (Zarit Burden Interview and Parkinson’s Disease Questionnaire-Carer).

**Results:**

Data collection was completed in February 2024, with 28 participants finishing the study (intervention group: n=15, 54% and control group: n=13, 46%). Data analysis is underway, with results expected to be published in December 2024.

**Conclusions:**

This study aims to provide significant insights into the effectiveness of dance or music and meditation in improving the quality of life and slowing the progression of PD. The findings are anticipated to support using these nonpharmaceutical therapies as complementary approaches to managing PD.

**Trial Registration:**

CTRI/2023/03/051064; https://tinyurl.com/2xdus53j

**International Registered Report Identifier (IRRID):**

DERR1-10.2196/59018

## Introduction

### Background

Parkinson disease (PD) is the second most common neurodegenerative disease, with over 10 million people worldwide having PD [1]. PD is highly prevalent in Asia, with a frequency of 70 patients with PD per 100,000 people in India [2,3]. PD is a progressive, multidimensional disorder characterized by the degeneration of dopaminergic neurons, resulting primarily in motor dysfunctions including bradykinesia, rigidity, tremors, and postural instability [4]. PD can also result in concurrent nonmotor manifestations including psychiatric and mood-related alterations such as apathy, depression, and anxiety [5,6]. Cognitive involvement is prevalent as well, with a high occurrence of slow processing speed and deficits in learning and attention in patients with PD [7,8]. They are also seen to experience behavior disturbances [9].

Previous research suggests that medical or surgical treatments do not change the progression of PD [10]. It is seen that alternative therapies such as music, dance, and mindfulness might help delay the progression of PD symptomatology while improving the quality of life (QoL) [11,12]. In addition, therapies such as exercise and aerobic dance can have neuroprotective effects led by neuroplasticity [13]. Exercise is further seen to modulate dopamine neurotransmission [14].

### Music Therapy

It has been observed that music modulates the brain regions related to movement, behavior, and cognitive processes [15]. Musical rhythm offers time-based groupings of sounds that provide auditory cueing and signaling strategies. This can help in restoring internal synchronization mechanisms and enable easier control of motor rhythmicity [15]. Evidence suggests that music exposure can enhance balance and functional mobility in patients with PD [13]. Listening to different musical genres has also been shown to induce spatiotemporal sense and trunk oscillations in the gait of patients with PD [16]. The music further activates the amygdala-related pathways and is seen to reduce depression and elevate mood in patients with PD [17]. Abell et al [18] conducted a study demonstrating that choral singing for a minimum of 6 months led to improvement in both motor and non-motor symptoms in patients with PD.

### Dance Therapy

Dance therapy uses sensory-motor methods such as visual and spatial focus to facilitate improvement in one’s physical and mental health [11]. Dancing entails the practice of movements, postures, and body control, which might address bradykinesia, postural instability, and rigidity associated with PD [19]. McNeely et al [20] conducted a 2-year longitudinal study, wherein significant improvements in motor measures were observed in weekly tango dance sessions, reflected through the assessments of the outcome measures at 12 and 24 months. Dance is also seen to enhance mood, socialization, self-esteem, and QoL in patients with PD [21]. Additionally, high levels of commitment are observed in training programs offering American tango versus traditional exercise [22]. Dance programs among patients with PD can further improve the QoL of caretakers [22].

Certain dance forms and particular cultural dances are seen to have varied influences on PD symptoms [23,24]. For instance, a study claims that ball dancing might be more effective than tango [23]. Furthermore, there is evidence that Amazonian dances have distinct cultural peculiarities such as knee flexion and accentuated hip movements that can improve PD motor symptoms [24]. However, there is limited research on the effect of Indian art forms on PD symptomology and progression.

### Meditation

Meditation is a mental state of quiescence that affects the body's functioning and helps patients gain attentional control and maintain focus on their intentions rather than being controlled by external environmental factors [25,26]. Mindfulness meditation stimulates the parasympathetic nervous system of the body through deep breathing exercises and positively impacts concentration and emotion regulation [27]. A mindfulness meditation research study conducted in South Korea, among patients with depression, anxiety disorders, and drug abuse, showed that meditation has positive effects on emotional and psychological stability, depression, anxiety, and memory loss [28].

Most of the studies to date have either been conducted over a short duration of time or on a small group of patients or have evaluated limited parameters. There has been no comprehensive review exploring the effect of dance or music and meditation on QoL, and limited studies take the QoL of caretakers into consideration. Additionally, there is limited research on the effectiveness of alternative therapies on the Indian population. This study aims to evaluate the effectiveness of music or dance and meditation on QoL and the progression of symptoms in patients with PD.

## Methods

### Study Design

**Figure 1:**
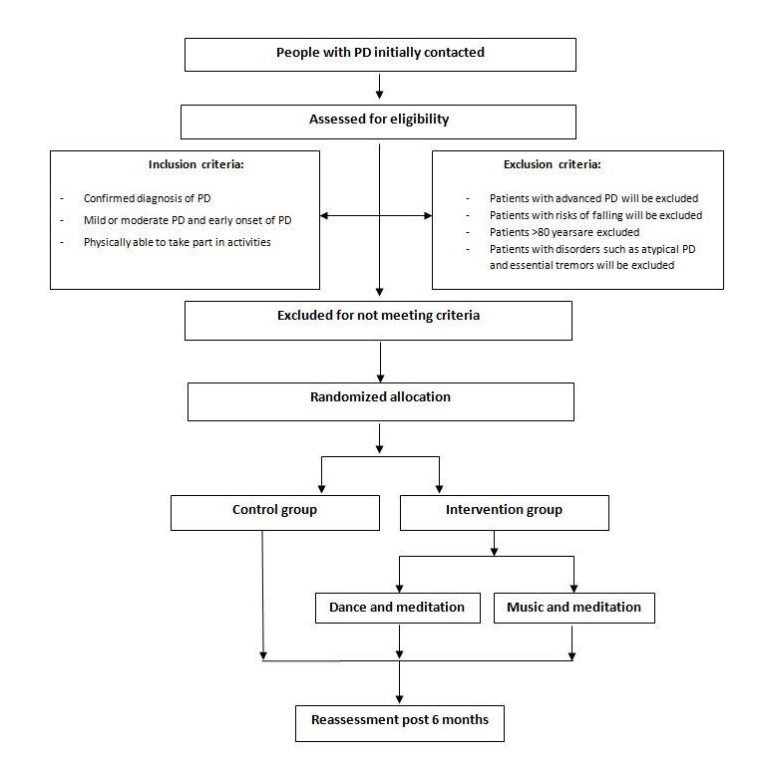
CONSORT (Consolidated Standards of Reporting Trial) flowchart summarizing the format of the study. PD: Parkinson disease.

The study is a single-blinded, longitudinal, randomized controlled trial (RCT) that will take place over a 6-month time period at Jaslok Hospital and Research Center, Mumbai, India. Participants will be randomly allocated into 2 groups: the control and intervention groups, having 15 participants in each arm. The intervention group will be further divided into dance or music as per the participant’s choice. The CONSORT (Consolidated Standards of Reporting Trial) flowchart summarizes the enrollment and allocation of the participants (Figure 1).

### Aims

#### Primary End Point

The primary end point is to examine the effect of dance or music and meditation on the progression of mild and moderate PD using the Unified Parkinson’s Disease Rating Scale (UPDRS) I-III and Parkinson’s Disease Questionnaire-39 (PDQ-39) scores in patients with PD.

#### Secondary End Point

The secondary end points include the following.

- Detecting the impact of these therapies on cognition, mood, and behavior through neuropsychological batteries.
- Assessing the impact on the QoL of the caregivers of patients with PD.

### Participants

#### Inclusion Criteria

The inclusion criteria are as follows:

- Patients with a confirmed diagnosis of mild to moderate PD (motor UPDRS III score of 43 or below)
- Patients should be residents of Mumbai (the city where the research is conducted)
- All patients should be physically able to take part in the activities

#### Exclusion Criteria

The exclusion criteria are as follows:

- Patients with advanced PD
- Participants with extreme balance-related problems or the inability to move
- Age >80 years
- Patients with other movement-related disorders such as atypical Parkinsonism and essential tremors
- Patients who underwent deep brain stimulation
- All patients with secondary Parkinsonism
- Patients with a history of falls

### Randomization

Eligible participants will be randomized into the intervention and control groups through a randomized allocation sequence devised by an independent statistician.

### Blinding

This is a single-blinded study, as the assessors measuring the outcomes will be blinded to the allocation of groups.

### Assessments

All assessments will be conducted during the “on phase,” that is, during the phase when the PD medication is in effect. The outcomes will be assessed by a specialist nurse, a neuro-consultant, and a consultant psychologist. The neuro-consultant is a functional neurosurgeon with pioneering work in this field and more than 30 years of experience in movement disorder surgeries. The specialist nurse has been involved in the treatment of movement disorder patients for more than 26 years and is a certified UPDRS assessor. For the reassessments, the marker who conducted the initial assessment will conduct subsequent assessments.

### Attrition and Missing Data

Due to the long duration of the study, it is expected that there will be a moderate attrition rate. To reduce the attrition rate and maintain commitment to the project, the importance of complete participation and consistency during the sessions will be conveyed to the team members and participants. The reassessments will be arranged at the participants’ convenience to minimize the possibility of missing data.

### Intervention Group

The patients will be given the option to choose from dance and music modalities. Meditation will be provided in every session. Active engagement for therapy is required for up to 6 months. All the activities will be improvised as per the abilities of the patients. The duration of each class will be around 1 hour. Both music and dance modalities follow a similar structure. All the classes will be followed by a 3-part format.

- Part 1: Classes will begin with 10 minutes of warm-up activities that will cover a wide range of stretching and hand-leg-eye coordination exercises.
- Part 2: The 45-minute dance or music sessions will follow a different format during this stage.
- Part 3: The sessions will conclude with 15 minutes of guided meditation.

The dance sessions will begin with warm-up activities with a focus on Indian classical dance steps. The classical dance focuses on gait and balance-related tasks, intending to reduce bradykinesia, shuffling gait, and the tendency to freeze in PD [29]. Classical dances emphasize greatly on hand-motor coordination and eye movement [29,30]. The eye-movement exercises might further improve lower gaze palsy in patients with PD. Alongside this, the patients will be engaged in the dance form of their choice. They will be offered various options of dance forms such as Bollywood dance, group dance, and garba. Patients can follow the choreography of the dance invigilator or perform freestyle. Following this provided that every session has at least 2 couples, the patients will practice waltz for 10 minutes.

Meanwhile, in the music sessions, the patients will engage in vocal exercises and will be given the choice of playing a musical instrument; singing the karaoke; or auditory stimulation, that is, listening to the karaoke. They will be encouraged to actively participate in the sessions.

The meditation will be administered by a professional trained in conducting meditation classes for the general population. Participants will be asked to close their eyes and sit in a relaxed posture either cross-legged or on a chair. Hands will be on knees with palms open. The mindfulness meditation will include guided instructions for deep breathing followed by a body scan and then guided instructions to pay attention to their thoughts and feelings.

All participants will be instructed to follow their daily routine and normal medication schedule. They will be instructed to keep the program coordinator informed if they make any significant changes to their treatment or their medication dosage or schedule. The participants in the intervention group will be asked to commit to the program and to take part in music or dance and meditation sessions thrice, weekly, for 6 months. On a weekly basis, patients should attend at least 1 session in physical presence, while the other sessions will be monitored through videoconferencing and home-based logs.

The patients will also be provided with the recordings of videos with instructions and a breakdown of each activity taught during the face-to-face sessions, making it easier for the participants to practice at home. A complete audio of the guided meditation schedule will be shared with the patients as well. The patients will be instructed to maintain an activity logbook documenting their adherence to home-based practice. Class attendance will be monitored and documented.

### Control Group

Participants in the control group will be instructed to continue their regular activities and medication during the study period. They will be offered the chance to enroll in the dance or music sessions after the completion of the study. During the study, the patients from the control group will not interact with participants in the intervention group.

### Reassessments

Reassessments for all participants will take place within 6 months of allocation (this is a deviation from the originally submitted protocol, which had assessments at 2 time points: 3 and 6 months. The deviation was incorporated considering the fact that major changes were likely to be noted in only at 6 months).

### Outcomes Measures

#### Primary Measures

##### Progression of PD Symptoms

The UPDRS I-III consists of 31 items assessing the severity of nonmotor and motor aspects of daily living and motor examination [31]. The responses will be scored from 0=no impairment to 4=severe impairment. Higher scores indicate higher severity (maximum score=124). This measure has demonstrated a strong internal consistency with α=0.96 and high concurrent validity against other similar constructs [31].

##### Quality of Life

The PDQ-39 is a 39-item self-report measure that measures the functional impairments in the following modalities: Parkinsonism symptoms, systemic symptoms, and emotional and social functioning [32]. Each item will be scored on a categorical scale from 0=never to 4=often. A summary index is further calculated ranging from 0 to 100. Higher scores indicate worse QoL. The PDQ-39 is widely used with good internal consistency of α=0.72-0.93 [33] and good concurrent validity against the Hoehn and Yahr Index and other similar constructs (0>0.60) [34].

#### Secondary Measures

##### Mood

The Beck Depression Inventory is a 21-item measure that is used worldwide to assess the severity of depression [35]. The items can be grouped into distinct symptoms of depression: cognitive, somatic, affective, and vegetative symptoms. This scale has a strong internal consistency of 0.88 and strong concurrent reliability with the depression subscale of the Symptom Checklist-90-R [36].

Additionally, the Parkinson Anxiety Scale is a 12-item measure that assesses 3 categories of anxiety: persistent anxiety, episodic anxiety, and avoidance behavior [37]. Each item will be scored on a categorical scale from 0=never to 5=often. Higher scores indicate worse anxiety (maximum score=63). This measure has a strong internal consistency of α=0.87-0.89 and good known-group and convergent validity [37].

##### Balance and Mobility

The timed up and go (TUG) and Berg Balance Test (BBT) assess mobility and risk of falls. The TUG is an observational test testing the gait, style, and time taken for the participant to get up from a chair, move for 3 meters, turn around, and return to the seat [38]. The TUG is seen to have good retest and interrater reliability in patients with PD [39]. Additionally, the BBT is an assessor-rated scale that records mobility through 14 tasks, with their performance being rated on a 5-point scale ranging from 0=poor performance to 4=good performance [40]. The BBT has a good internal consistency of α=0.93 and strong concurrent validity as strongly correlated with the Mini Balance Evaluation Systems Test [41].

##### Caregiver Scale

The Zarit Burden Interview is a 29-item self-report measure assessing the burden of the caregiver, measuring the psychological well-being, financial, and overall burden [42]. Each item is to be scored from 0=never to 4=always. The Zarit Burden Interview has good reliability with α=0.89-0.95 and good external validity with PD-related carer scales [42]. The Parkinson’s Disease Questionnaire-Carer is a 29-item measure further assessing the mood, social life, and relationships of the caregiver with a good test-retest reliability [43]. This scale will be rated from 0=never and 4=always. Higher scores indicate worse QoL (maximum score=100).

##### Cognition Scale

The Mini-Mental State Examination evaluates performance at various cognitive components including orientation, attention, and recall [44]. A higher score signifies better cognitive abilities (maximum score=30). This measure has good test-retest reliability (0.80-0.95) and satisfactory construct validity against various gold standards used to diagnose cognitive impairment [44].

##### Behavioral Disorders

The Questionnaire for Impulsive-Compulsive Disorders in Parkinson’s Disease Rating Scale is a 28-item scale that records impulsions, compulsions, and compulsive medication usage [45]. Responses will be recorded on a 5-point Likert scale with 0=Never and 4=Often. This measure demonstrated high sensitivity and specificity across all subscales [45].

### Data Analysis

IBM SPSS software will be used for the statistical analysis. Descriptive statistics will summarize the demographics, health conditions, and clinical outcomes at all 3 time points. Categorical variables including the Likert rating scales will be expressed in percentages and frequencies. The median and mode will also be ascertained for these data. Additionally, chi-square tests will be conducted, and *P* values will be ascertained to decipher the difference between the 2 groups against each outcome variable throughout the 3 time points.

### Ethical Considerations

The study was approved by the scientific committee of the Jaslok Hospital and Research Centre (vide letter dated February 18, 2023; reference: -EC/1156/2023; Amendment 1). It was further approved by the ethics committee of Jaslok Hospital (vide letter dated February 18, 2023). It was also registered with Clinical Trials Registry India (dated March 27, 2023; CTRI/2023/03/051064). All participants provided informed consent (Multimedia Appendix 1). All data will be anonymized. The participants were provided travel compensation ($200 US per participant).

### Expected Outcomes

Participants in the intervention group are anticipated to show significant improvements in both motor and nonmotor aspects of PD. Improvements in gait and balance are expected, alongside positive changes in QoL, mood, cognition, and behavior. Caregivers of participants in the intervention group are also likely to report an enhanced QoL due to the improved condition and mood of the patients they care for.

## Results

The data collection for this paper commenced in February 2023 and was completed in February 2024. A total of 30 participants were recruited, of which 28 completed the study (intervention group: n=15, 54% and control group: n=13, 46%). The data are currently being analyzed, and the final results are anticipated to be published in December 2024.

## Discussion

### Expected Findings

This RCT explores the effects of dance, music, and meditation on functional mobility, balance, QoL, cognition, mood, anxiety, behavior, and caregiver burden in individuals with PD. To date, there has been no research comprehensively investigating the benefit of dance or music and meditation on the QoL of patients with PD or the caretakers in Asian populations (including Indians). This will be the first study of its kind to be conducted in an Indian setup. It is also to be noted that none of the earlier studies offered the participants the choice to choose a modality of their preference. This will be the first paper that allows the participant to choose from music and dance.

The RCT, including 10 patients with PD, by Duncan et al [13] aimed to examine the effects of a 2-year, community-based dance class on disease severity and functional mobility in patients with PD. Assessments at baseline, 12 months, and 24 months showed that the intervention group had lower scores on the Movement Disorder Society-UPDRS III, Mini Balance Evaluation Systems Test, and other measures than the control group. The study concluded that participating in community-based dance classes over 2 years improved motor and nonmotor symptom severity, activities of daily living, and balance in individuals with PD [13].

The recent study by Hashimoto et al [46] involved 46 patients with mild to moderate PD who participated in a 12-week intervention. The patients were divided into a dance group, a PD exercise group, and a nonintervention group. The primary outcome measures included the TUG and BBT for motor assessment, the Frontal Assessment Battery at the bedside and Mental Rotation Task to assess cognitive function, the Apathy Scale and Self-rating Depression Scale to assess mental symptoms, and the UPDRS for general assessment. The results showed that the dance group exhibited significant improvements in all the outcome measures, highlighting dance’s positive impact on the mental well-being, self-esteem, and social engagement of those with PD [46]. The guided meditation component may further contribute to stress reduction and relaxation.

The study by Katlen da Silva et al [12] aimed to evaluate the effects of music-based physical therapy on individuals with PD in terms of muscle strength (Medical Research Council Test and Sitting-Rising Test), cognition (Trail Making Test), muscle strength, balance (BBT), and functional mobility (TUG). It was a controlled, nonrandomized clinical trial with an A-B-A design involving 13 individuals with PD. The results showed that music-based physical therapy helped improve balance and functional mobility in individuals with PD. However, these improvements were not maintained after the therapy was discontinued.

The study by Lihala et al [47] in India examined the impact of dance therapy on cognition, QoL, and motor symptoms in 10 patients with mild-to-moderate PD. The results showed improved cognitive function and QoL, as indicated by increased Montreal Cognitive Assessment scores and decreased PDQ-39 scores [47].

Lee et al [48] examined the effects of virtual reality dance exercises on patients with PD. They found that after 6 weeks of treatment, the experimental group showed significant improvements in BBT (from mean 46.0, SD 1.3 to mean 48.1, SD 3.0); activities of daily living (from mean 87.9, SD 1.4 to mean 91.1, SD 3.0); and Beck Depression Inventory (from mean 20.4, SD 0.9 to mean 18.2, SD 2.0) compared to the control group, highlighting a positive impact on these aspects for patients with PD [48].

The studies mentioned above highlight that dance, music, and mindfulness have improved motor and nonmotor symptoms, activities of daily living, balance, cognitive function, and QoL in patients with PD.

### Limitations and Future Implications

However, the study might be subject to some limitations. The 2 limitations to consider are the small sample size and the short duration of the study. A 6-month timeframe may not be sufficient to observe changes in nonmotor symptoms. Future larger, longitudinal RCTs are warranted to verify results. Motor and nonmotor symptoms of PD such as motor disability, gait and balance difficulties, speech disability, and pain are likely to hinder participation in activities such as dance and music. To compensate for this, each session will be customized according to the participants’ needs and abilities. Additionally, the results might be confounded by factors such as changes in medication or differences in baseline medication taken by the patient. Therefore, any changes made to lifestyle and medication over the course of the study will be constantly monitored. This paper will build upon previous research and delve into the effectiveness of non-pharmaceutical therapies in possibly delaying the progression of PD.

### Conclusions

This study aims to provide significant insights into the effectiveness of dance or music and education in improving the QoL and slowing the progression of PD. The findings are expected to support the use of nonpharmaceutical therapies as a complementary approach to managing PD, offering potential benefits to both patients and their caregivers.

## Appendix

Multimedia Appendix 1 A consent form.

## Abbreviations

BBT: Berg Balance Test
CONSORT: Consolidated Standards of Reporting Trial
PD: Parkinson disease
PDQ-39: Parkinson’s Disease Questionnaire-39
QoL: quality of life
RCT: randomized controlled trial
TUG: timed up and go
UPDRS: Unified Parkinson’s Disease Rating Scale

## Data Availability

The datasets generated during or analyzed during this study will be available from the corresponding author on reasonable request.
