# Supplementary material for: Impact of Dance or Music and Meditation on the Progression of Parkinson Disease With Mild or Moderate Severity: Protocol for a Pilot Randomized Controlled Trial
Source: JMIR Res Protoc. 2024 Oct 29;13:e59018. doi: 10.2196/59018 (PMC11558214; doi:10.2196/59018)
Supplement: Multimedia Appendix 1 [file resprot_v13i1e59018_app1.pdf]

INFORMED CONSENT FORM

PART I: Information Sheet

PROJECT TITLE

Impact of dance/music and meditation on progression of Parkinson's disease (PD) with mild and moderate severity. (PILOT STUDY)

This is a Pilot study and does not impact clinical decision making regarding medical or surgical management of the disease. The study will correlate the impact of dance/music and meditation on the progression of Parkinson's disease of mild to moderate severity. The purpose of the study is to evaluate the effect of above modalities on the Parkinson's disease and the quality of life of the patients and the caregivers as well as the effect on the mood and behavior of the patients.

PRINCIPAL INVESTIGATOR

Dr. Paresh Doshi
Department of Neurosurgery
Jaslok Hospital and Research Centre, Mumbai
Contact No. 9930960960

PROCEDURE/INVESTIGATIONS IF ANY TO BE PERFORMED - Nil

FORESEEABLE RISKS AND DISCOMFORTS ADEQUATELY DESCRIBED AND WHETHER PROJECT INVOLVES MORE THAN MINIMAL RISK. - Nil

BENEFITS TO PARTICIPANT/PATIENT, COMMUNITY OR MEDICAL PROFESSION AS MAY BE APPLICABLE

The data collected from the study will be utilize to see the benefits of dance/music and meditation on the progression of Parkinson's disease and their impact on mood, behavior and the quality of life of the patients which will help the community in future.

POLICY ON COMPENSATION

The Hospital does not make any provisions to compensate you for study related injury.

AVAILABILITY OF MEDICAL TREATMENT FOR SUCH INJURIES OR RISK MANAGEMENT

As this is an observational study, there will not be any deviation from the standard treatment protocol. The patients will perform the activities under supervision.

ALTERNATIVE TREATMENT IF AVAILABLE – Not applicable to our study (Pilot study)
COST OF THE TREATMENT – No additional cost will be charged from patients for participation

CONFIDENTIALITY

Your personal data shall be confidential. You consent for the doctors or the institute to use your medical details, details of this therapy for academic research purpose, without disclosing your personal details.

VOLUNTARY PARTICIPATION

Your participation in this study is entirely voluntary. At any stage you may choose not to continue the therapy.

WITHDRAWAL FROM THE TREATMENT

Your Participation in this study is entirely voluntary. You may choose to withdraw from this study any time.

CONTACT DETAILS OF CHAIRMAN OF THE IEC (INSTITUTIONAL ETHIC COMMITTEE) FOR APPEAL AGAINST VIOLATION OF RIGHTS

Mr. Ashok Vyas - Phone No. 02266573007

INDEMNITY

You have read the above / have been explained the same in a language that you understand, and hereby give your consent to proceed with the study. I understand that the information collected during my participation in this research and sections of any of my medical notes may be looked at by responsible persons (ethics committee members / regulatory authorities). I have been informed my regular medical management will be same during the study. There is no additional cost added to my treatment for this study purpose. I give access to these individuals to have access to my medical records. I understand that there will be no deviation in the treatment protocol for the study purpose. I understand that my participation is voluntary and there will be no change in my treatment protocol by either denying/accepting the participation.

CONTACT PERSON:

If you have questions about the treatment or your rights as participants, you can call the institutional review board, which is the committee that reviewed and approved this treatment. Dr Fazal Nabi- Secretary - Ethics Committee, Jaslok Hospital and Research centre. **Contact 9820854433**.

PART II

Certificate of Consent

Jaslok Hospital and Research Centre, Mumbai

Subject Particulars

Name

Address

Sex- Female/Male

Age

I have read the foregoing information, or it has been explained to me in a language I understand. I have had the opportunity to ask questions about it and any questions that I have asked have been answered to my satisfaction. I consent voluntarily to participate as a participant in this study.

Signature/ Thumb print of Patient ________________

Date __________________

Witnessed By _______________

STATEMENT BY THE RESEARCHER/PERSON TAKING CONSENT.

I have accurately read out the information sheet to the potential participant/Patient, and to the best of my ability made sure that the participant understands that the following will be done:

1. Dance/Music and meditation acts, one session/week in hospital and remaining week at home.

2. Various score assessments at the end of 3 and 6 months.

3. No deviation from the standard aspects of care and treatment.

I confirm that the participant was given an opportunity to ask questions about the study, and all the questions asked by the participant have been answered correctly and to the best of my ability. I confirm that the individual has not been coerced into giving consent, and the consent has been given freely and voluntarily.

A copy of this ICF has been provided to the participant.

Name of Researcher/person taking the consent______________________

Signature of Researcher /person taking the consent________________________

Date _________________________
